# The facilitators and barriers of mHealth adoption and use among people with a low socio-economic position: A scoping review

**DOI:** 10.1177/20552076231198702

**Published:** 2023-09-05

**Authors:** Tessi M Hengst, Lilian Lechner, Daan Dohmen, Catherine AW Bolman

**Affiliations:** 1 Department of Psychology, 10198 Open University of the Netherlands, Heerlen, Netherlands; 2 Department of Management Science, 10198 Open University of the Netherlands, Heerlen, Netherlands

**Keywords:** mHealth, adoption, barriers, facilitators, scoping review, SEP, socio-economic position

## Abstract

**Background:**

Despite the fact that 95% of the global population has a mobile phone, the adoption of mHealth lags among people with a low socio-economic position (SEP). As they face health risks and many barriers in the traditional offline healthcare system, mHealth has an important role. Therefore, it is important to understand the factors that promote and impede mHealth adoption among people with a lower SEP.

**Objective:**

The current study aims to provide an overview of what is known about the facilitators and barriers to the adoption and use of autonomous mHealth applications among people with low SEP.

**Methods:**

A PRISMA scoping review in which the scientific databases PubMed, Web of Science, PsychInfo and SocINDEX were searched in the period of March 2017 to March 2022.

**Results:**

Of the 1827 indexed papers, 13 papers were included in the review. In these papers, 30 factors have been identified as promoting or hindering the adoption of autonomous mHealth applications among low SEP people.

**Conclusions:**

Thirty factors were found to facilitate or impede mHealth adoption among people with a low SEP, categorised into intrapersonal, interpersonal, community, ecological and app specific levels. Factors are assumed to be interrelated. The relationship between traditional (offline) care and digital care appeared to be of particular interest as the current study revealed that face-to-face contact is a prerequisite of mHealth adoption among people with low SEP. Therefore, a well-structured cosmopolitan system of stakeholders has been recommended.

**Trial registration:**

This study was registered in OSF (https://doi.org/10.17605/OSF.IO/ATU9D).

## Introduction

### Rationale

Due to the increased use of mobile technology, mobile health (i.e. mHealth) applications are increasingly being used in healthcare settings around the world.^1,2^ mHealth is defined as ‘a medical and public health practice supported by mobile devices, such as mobile phones, patient monitoring devices, personal digital assistants and other wireless devices’.^3^ mHealth, in turn, is used for healthcare delivery, self-monitoring of diseases, health education and promotion and behaviour change communications.^2^ It has the potential to differentiate itself from more traditional health services because it provides the opportunity to inexpensively share and exchange health-related information and guidance in a fast, efficient and effective manner which, in turn, could reduce costs and time while improving the access to healthcare.^3,4^

Hence, mHealth applications are increasingly being used to reach even the hard-to-reach populations^5^ as they potentially could overcome geographic, socio-economic, linguistic and cultural barriers^6,7^ and therewith make healthcare more accessible to these populations.^8^ However, despite people's attitudes toward mHealth being positive,^9^ research shows that the adoption (i.e. the acceptance of the technology) of mHealth lags among people with lower levels of education,^10–12^ a lower income^1,10–12^ and ethnic minorities.^7,12–14^ As discussed by Stowell and Lyson,^15^ these are the vulnerable groups who face health inequalities and are considered as having a low socio-economic position (SEP). Thus, people with a low SEP appear to have a lower adoption of mHealth than those with a high SEP.^9,11^

This phenomenon is often referred to as the ‘digital divide’, with some people benefiting less from electronic health technology than others. People with a low SEP often face a digital divide.^13,14,16^ Earlier, the digital divide constituted the inequality between people based on their access to the Internet (i.e. the first-level digital divide). However, current concerns are raised about the inequalities regarding digital literacy and skills (i.e. the second-level digital divide) and the difference in the way technology is used (i.e. third-level digital divide).^12,16^ On the one hand, people living in poverty have increasing access to mobile phones, and thus to mHealth, which may reduce health disparities.^3^ On the other, existing health inequalities could be magnified by this digital divide as people with a low SEP are more affected by the second- and third-level divide than people with a high SEP.^1,11,14^

In addition, people with a low SEP experience a number of barriers in the healthcare system, such as language barriers, a lack of financial resources and reduced access to health insurance.^6,13^ Moreover, psychosocial barriers are faced, such as a lack of trust in the healthcare system^4,14^ or a lower health literacy (i.e. the ability to seek out, find, evaluate and appraise, integrate and apply what is learned in online environments to solve a health problem) resulting in people with a low SEP searching for less information online.^16^ Furthermore, cognitive factors play a role. Despite the fact that people with a low SEP often face greater health risks than their higher SEP counterparts,^5,9,17^ they often have lower motivation to live a healthy lifestyle and work with health-related tools,^12^ whilst they have a lower self-efficacy for behaviour change^5^ or eHealth use.^9^

Hence, people with a low SEP have a need for health and medical care, but it seems that the healthcare system might not connect well enough – either in person or via mHealth – with the motivation and capabilities of people with a low SEP. Thus, there is a strong need for providing health information and healthcare through mHealth in a way that can be embraced by people with a low SEP. Not only could this increase access to health care for people with a low SEP, it could also provide a solution for vulnerable people who might prefer avoiding public spaces such as the hospital because of their medical state (e.g. patients with COPD or an impaired immune system).

By increasing the accessibility and ease of use of mHealth, its potential to reach those with lesser access to health systems in terms of service delivery, health workforce, health information, medical products, vaccines and technologies (i.e. the World Health Organization's six building blocks for a strong health system^18^); would be optimised. Herewith, health information provision and health-related services could be enhanced to support effective and accessible prevention and care rather than perpetuate existing health disparities.^14^

### Objectives

As mHealth applications have the potential to enable health professionals and researchers to tailor health-related content to the user characteristics, contexts, wants and needs,^17^ it is important to fully understand the factors that promote and impede the adoption of mHealth applications among the lower SEP group so that the access to and use of (preventive) healthcare for these groups can be increased to ensure that health disparities are reduced.

To date, however, an overview of the facilitators and barriers of the adoption of mHealth applications among people with a low SEP is lacking. The existing literature consists mostly of single studies focusing on a particular application, a particular (vulnerable) target group, or a specific lifestyle behaviour, thus missing the overall insights with regard to the low SEP target group. Hence, by answering the research question *What facilitators and barriers promote and impede the adoption of mHealth applications among people of a lower socio-economic position?* The current scoping review aims to combine insights gained from studies on specific applications, target groups and lifestyle behaviours by providing an overview of what is known about the factors influencing the adoption of mHealth applications and where knowledge gaps remain. Hence, a scoping review is conducted to systematically map and summarise the theoretical insights gained by the research done in this area as well as to identify the knowledge gaps with regard to mHealth adoption by low SEP groups.

Accordingly, the barriers and facilitators are categorised based on the classification of health behaviour theories as discussed by Stowell and Lyson.^15^ This classification is comparable to the social-ecological model developed to enhance the interrelations between personal and environmental factors. Within the study of Stowell and Lyson,^15^ this classification has been applied to the design and evaluation of mHealth interventions for vulnerable populations. The model encompasses an intrapersonal, interpersonal, community and ecological level. On an intrapersonal level, the factors within the individual are considered, such as skills, behaviour, beliefs and knowledge. Accordingly, the interpersonal level looks at the influence of others on the individual (e.g. the relationship with family, friends or carers). The community level focuses on the physical and social environment including the influence of societal relationships, external companies (e.g. larger organisations or the government) and residential areas on the individual. Finally, there is an ecological level, which looks at the influence of the combination of the previously mentioned factors along with the influence of laws, regulations and policies. A fifth ‘app specific’ level was added by the current study's authors to accommodate the facilitators and barriers specifically related to the mHealth applications. This does not include (inter)personal, corporate, or external factors, but includes characteristics of the application such as the design or functionalities.

## Methods

Within the current study, mHealth adoption is referred to as the willingness for, acceptance of, and use of mHealth. To research this topic, a systematic scoping review was performed as described in the PRISMA guidelines for scoping reviews.^19,20^ The PRISMA-ScR checklist can be found in Appendix A.^20^ The model considering population, intervention, comparator and outcome of interest (PICO) was followed to define the searching strategy and thereby the inclusion and exclusion criteria.

### Search strategy, eligibility criteria and study selection

With regard to the information sources, the scientific databases PubMed, Web of Science, PsychInfo and SocINDEX were used as these databases include medical, psychological and sociological papers. Several filters were applied: papers needed to be published in the last five years (i.e. between March 2017 and March 2022), needed to be published in English and needed to have a full-text available to be included in the study. A timeframe of five years has been chosen because developments in mHealth are moving incredibly fast. Hence, factors that influenced the adoption of mHealth applications ten years ago may now be less influential or not at all (e.g. access to the Internet). Therefore, we chose to examine the most recent papers. Additionally, for text availability, we chose to filter by full text so that all articles that were shown had a link to the full text available.

Accordingly, a search strategy was developed. This search strategy was peer reviewed by a bibliometric information specialist and three co-researchers. After having done so, the search string was entered into PubMed (see Table 1). Afterwards, this search string was modified to the other databases, after which the search was performed in the Web of Science, PsychInfo and SocINDEX database. Finally, an additional search was conducted in Google Scholar to ensure that no relevant scientific articles had been overlooked and that any important books and grey literature were included in the search. Based on this, one article from the ACM Digital Library was added. With this, several types of literature (i.e. scientific articles, pre-publications, working papers, books, citations, patents and legal documents) have been considered within the scoping review.

**Table 1.** Search strategy for the PubMed database.

Building block	Search string
Mobile health technology	(((eHealth[tiab] OR e-Health[tiab] OR mHealth[tiab] OR m-Health[tiab] OR “mobile health”[tiab] OR “digital health”[tiab] OR telehealth[tiab] OR “health communication*”[tiab] OR (telemedicine* NOT (telepathology OR teleradiology OR telerehabilitation)) OR “health game*”[tiab] OR “health communication”[tiab]) AND (“technolog*”[tiab] OR “technology-facilitated”[tiab] OR “technology based”[tiab] OR “technology-based”[tiab] OR “communication technolog*”[tiab] OR “mobile communication”[tiab] OR “telecommunication*”[tiab] OR “internet based*”[tiab] OR “internet-based*”[tiab] OR app[tiab] OR apps[tiab] OR “mobile communication”[tiab] OR “web communication*”[tiab] OR technolog*[tiab] OR application*[tiab] OR software[tiab] OR “tool*”[tiab])) OR (“health tool”[tiab] OR “health technology”[tiab] OR “health app”[tiab] OR “health application*”[tiab]))
Barriers or facilitators	AND (((barrier*[tiab] OR facilitator*[tiab] OR promot*[tiab] OR imped*[tiab] OR obstruct[tiab] OR hindrance[tiab] OR interfer*[tiab] OR facilitat*[tiab] OR stimulat*[tiab] OR determinant*[tiab]) OR (“support*”[tiab] OR “aid”[tiab] OR aiding[tiab] OR “assist*”[tiab] OR “help*”[tiab])) OR (“Digital inclusion”[tiab] OR “digital involvement”[tiab] OR “digital divide”[tiab] OR “health services accessibilit*”[tiab] OR “marginalization”[tiab] OR “digital inclusiv*”[tiab] OR “social health inequality*”[tiab]))
Vulnerable people	AND ((“vulnerable people”[tiab] OR “vulnerable citizen*”[tiab] OR “vulnerable population*”[tiab] OR “underserved population*”[tiab] OR “disadvantaged population*”[tiab] OR “disadvantaged group*”[tiab] OR “deprived population*”[tiab] OR “social deprivation”[MeSH] OR “underprivileged population*”[tiab] OR “sensitive population*”[tiab]) OR (“low socioeconomic position*”[tiab] OR “low socio-economic position*”[tiab] OR “low social class”[tiab] OR “low social status”[tiab] OR poverty[tiab] OR unemployed[tiab] OR unemployment[tiab] OR unemployment[MeSH] OR “social inequalit*”[tiab] OR “social inequit*”[tiab] OR “social disparit*”[tiab] OR “health status disparit*”[tiab] OR “health disparit*”[tiab] OR “healthcare disparit*”[tiab] OR “ethnic minorit*”[tiab] OR “racial minorit*”[tiab] OR “socially disadvantaged group*”[tiab] OR “psychiatric problem*”[tiab] OR “psychiatric disorder*”[tiab] OR addiction*[tiab] OR “addictive behavior”[tiab] OR “substance dependence”[tiab] OR “intellectual disabilit*”[tiab] OR “mental disabilit*”[tiab] OR “mental retardation”[tiab] OR “intellectual development disorder”[tiab] OR idioc*[tiab] OR “psychosocial mental retardation”[tiab] OR “mental deficienc*”[tiab] OR “limited cognitive capacity*”[tiab] OR “disabled persons*”[tiab] OR “low health literacy”[tiab] OR “low digital literacy”[tiab] OR “low computer literacy”[tiab] OR “low literacy group*”[tiab] OR “low-literacy group*”[tiab] OR “older age”[tiab] OR “older adult*”[tiab] OR elderly[tiab] OR “chronic disease”[tiab] OR multimorbidit*[tiab] OR “multi-morbidit*”[tiab]))

The articles that emerged from the search were imported to the reference management tool EndNote (version 20). Then, duplicate records were removed according to the three-step de-duplication method discussed in the protocol study of Bramer et al.^21^ In the first step, the EndNote settings were changed for the tool to show page numbers. Import filters and output styles were downloaded (http://bit.ly/emcendnote) accordingly to make the page numbers display correctly. In the second step, the references were put into a temporary EndNote library, were exported with the downloaded export filter and were put in the final EndNote library. In the third and last step, duplicates were identified and removed.

The final scope (*N* = 1827) was then imported in the systematic review tool Rayyan.^22^ This tool provides an overview of the article’s title, abstract and keywords, which facilitates and speeds up the screening process. Subsequently, the screening process consisted of three phases.

First, the abstracts were screened by two independent screeners using the inclusion and exclusion criteria in Table 2. The abstracts on which the screeners did not agree were jointly discussed and rescreened to reach a consensus. A third researcher was available in case no agreement was reached, but this was not the case.

**Table 2.** The final inclusion and exclusion criteria using the PICO method.

PICO	Inclusion criteria	Exclusion criteria
Population	First screening: Vulnerable people (e.g. chronically ill, elderly, ethnic minority) Second screening: People with a low socioeconomic position, i.e. people with a low income or low-income countries, people with a low education attainment, people from ethnic minorities, or people from deprived countries	Studies that address the opinion or evaluation of other stakeholders, or healthcare workers specifically
Intervention	Autonomous mHealth applications that can be adopted by low SEP individuals on a mobile device (e.g. smartphone, tablet, computer) without requiring interaction with a third party (e.g. caregivers, relatives, other devices). Examples are as follows: Mental health applications Chronic disease management applications Self-monitoring applications	Medical applications focused on healthcare personnel or relatives of the low SEP individual. Interventions that are not autonomous but interact with other persons (e.g. caregivers) by, e.g. SMS, phone contact, instant messages, video calls, and social media. Studies focused on AR/VR/AI/robots Studies that focused on motion sensors or activity trackers. Feasibility-, cost-specific-, and protocol studies
Comparator	NA	NA
Outcome of interest	Factors that promote or hinder adoption. These may include (among others) psychosocial, technical, demographic, and environmental factors.	Studies that do not focus primarily on or explicitly address the factors that promote or hinder adoption.

Second, the inclusion and exclusion criteria were evaluated and tightened as a very large scope remained in the end (184 included papers). To prevent the search area from being too broad, which would result in the study results adding little value, it was decided to narrow the target group. This followed the procedure described in the pre-registration. Whereas facilitators and barriers were first sought for the broader target group of vulnerable people, this was further narrowed specifically to the group of people with a low SEP (see Table 2). Included, there should be studies on the factors known to influence mHealth adoption among the low SEP target groups, from as many different mHealth applications in as many countries as possible. As this scoping review aims to gain insight into the factors that promote or hinder the adoption of autonomous mHealth applications among people with a low SEP, the included articles should also be on applications that are used by and focused on lower SEP individuals (and not, for example, their carers). In addition, an autonomous application indicates that the application can be used independently by an individual. Articles about applications that use third-party interaction (e.g. through video calling, chatting or SMS) were therefore excluded. In line with that, applications that interact with external devices such as motion sensors or activity trackers were also excluded. Herewith, the focus is fully on the factors that relate to the adoption of mHealth applications that can be used for health-purposes by a low SEP individual without requiring interaction with third-parties or external devices.

Within the literature on mHealth applications, both individual studies as well as review studies on the facilitators and barriers to mHealth adoption among people with a low SEP, or among one of the three target groups (i.e. people with low income, people with low education, or ethnic minorities), are taken into account. All study designs were eligible to be included except for feasibility-, cost-effectiveness- and protocol studies. Feasibility studies often focus on a single application the results of which are often not widely applicable. Next, cost-effectiveness studies focus on costs rather than facilitators and barriers. Finally, protocol studies predominantly focus on the process rather than the outcome (i.e. barriers and facilitators) which is what we are interested in in the current study. With these criteria in mind the abstract of the articles in scope were screened again by two screeners (*N *= 452). A third independent screener was available if there were any doubts, but this was not the case.

Third, after the screening, the full text of the included articles was read. The articles that proved to be relevant were also screened on the reference list to see if there were any relevant articles among them, after which the full text of these relevant articles was also screened. This resulted in the final scope of 13 articles meeting the study requirements.

### Data collection and data synthesis

A data charting form was developed by three researchers to determine which variables should be included in the data charting table. Thereafter, the data were charted by one researcher and checked by a second researcher. An overview was made of the title of the paper, the author(s), the year of publication, the country of conducting the research, the research design, the target group, the sample size and the end users’ facilitators and barriers for mHealth adoption that were cited in the article (see Table 3). In this regard, based on the original authors’ conclusion it was decided whether something was categorised as a facilitator or barrier. Finally, the barriers and facilitators were categorised based on the classification of health behaviour theories as discussed by Stowell and Lyson.^15^ Table 4 provides an overview of the factors that were classified as one of the five levels. A thematic analysis was used in the classifications as many different terms were used in the articles for the same concepts (e.g. the terms ‘digital skills’ and ‘technical skills’ were used for the concept digital literacy).

**Table 3.** Overview of the relevant data of the included articles.

Interviews						
Title	Author(s)	Year	Target group	Study design	key facilitators	Key barriers
Attitudes and views on healthy lifestyle interventions for the prevention of dementia and cardiovascular disease among older people with low socioeconomic status: a qualitative study in the Netherlands	Eggink et al.	2021	Older adults with a low socioeconomic status	Semi-structured interviews	Tailoring, appropriate language use, consistent and trustworthy information, easy to use, and support	Limited faith in the coach and limited confidence in digital skills

Systematic reviews						
Title	Author(s)	Year	Target group	Study design	Key facilitators	Key barriers
Designing and evaluating mHealth interventions for vulnerable populations: a systematic review	Stowell et al.	2018	Low socioeconomic and racial/ethnic minority individuals	Systematic review – 83 included studies	Tailoring, familiar technology, frequent delivery of intervention content, personalisation of the timing, use of multimedia, variation, gamification, and confidentiality of information	Unfamiliar technology, lack of access to the technology, high costs, and low literacy
Barriers to the use of mobile health in improving health outcomes in developing countries: systematic review^1^	Kruse et al.	2019	Populations in developing countries	Systematic review – 30 included studies	Strong governance, commitment of the private sector, training on mobile devices, tailoring, policy reform, adapting and redesigning of intervention as technology advances, training of healthcare workers, partnerships with governments and nongovernmental organisations, and infrastructure.	Inadequate infrastructure, lack of equipment, technology gap, human resource issues such as a lack of availability training of health workers, time or work conflict, costs, lack of policies, literacy, language barriers, psychosocial issues such as personal stress, lack of training, concerns about privacy and confidentiality of information, lack of efficacy, and exposure to the intervention
Telehealth and indigenous populations around the world: a systematic review on current modalities for physical and mental health^a^	Dawson et al.	2020	Indigenous adults	Systematic review – 6 included studies	Evidence-based intervention, cultural tailoring, policies, and (indigenous) health professional involvement	Inadequate service reimbursement from third-party payers
Early patient-centred outcomes research experience with the use of telehealth to address disparities: Scoping review	Bailey et al.	2021	Populations at risk for disparities: racial and ethnic minority groups or people living in rural areas	Systematic scoping review – 41 included studies	Partnership with payers, access to technology, patient-centred design, cultural tailoring, delivering through trusted intermediaries, support staff available, confidential sharing of private information, personalisation of intervention components, reimbursement via payers, policies, and personalized human interaction	Inadequate access to technology, inadequate program supervision, lack of support staff available, poor integration of intervention in existing workflow, insufficient staff to deliver telehealth solutions, inadequate safeguards to ensure confidential data sharing, and inconsistent reimbursement coverage for providers.
Adaptation models, barriers, and facilitators for cultural safety in telepsychiatry: a systematic scoping review	Ruiz-Cosignani et al.	2021	Minority groups	Systematic scoping review – 58 included studies	Trained interpreters and healthcare providers, cultural tailoring, community involvement, user-centred design, pilot testing, evidence-based guidelines, multi-organisational collaborations and partnerships, and simplicity, availability and reliability of hardware, software and network	Mistrust, technical unsuitability of used devices, unreliable networks, low health literacy, limited awareness of the available services, work and family responsibilities, financial constraints, cultural gaps, and digital literacy
Barriers and facilitators in eHealth based lifestyle interventions for people with lower socioeconomic status: a scoping review of the current state	Al-Dhahir et al.	2021	People with a low socioeconomic status	Systematic scoping review – 59 included studies	Iterative intervention design, evidence-based intervention, concise and clear content, user friendliness and simplicity, interactivity, use of visual and multi-media elements, links to more information, tailoring, self-monitoring, social support, human coach, practical advice, reminders, trust, helping participants with technology use, incentives and rewards blended care, collaborating stakeholders, training healthcare professionals, collaborations with health insurances, and server support staff for technical issues	Technical challenges, amount of information or visuals, costs, frequency of reminders and messages, lacking infrastructure, limited digital literacy, language barriers, and lack of time

Mixed method studies						
Title	Author(s)	Year	Target group	Study design	Key facilitators	Key barriers
Can mHealth and eHealth improve management of diabetes and hypertension in a hard-to-reach population? – Lessons learned from a process evaluation of digital health to support a peer educator model in Cambodia using the RE-AIM framework^a^	Steinman et al.	2020	People living with non-communicable diseases in low to middle income countries	Mixed methods design: RCT combined with a mixed methods process evaluation	Strength and quality of the evidence of the intervention, its relative advantage, adaptability and triability, usability, mHealth as priority for leaders, cosmopolitanism of the systems, and stimulating external policies and incentives (such as cell phone coverage among patients and alignment of the intervention with international and national plans)	Lack of infrastructure, lack of equipment, psychosocial stressors, technology gap, mHealth not as a priority for peer educators, lack of policy, no coordination between mHealth and clinical workflows and systems, low education and literacy of users, and costs of mobile phones
Attitudes toward health, healthcare, and eHealth of people with a low socioeconomic status: a community-based participatory approach	Faber et al.	2021	People with a low socioeconomic status	Mixed method design: interviews combined with surveys and focus groups	Alignment with a person's attitude towards using the technology, stimulating personal communication, considering self-efficacy and perceived control, social support, tailoring, supportive health professionals, visuals, and meaningful and directly applicable feedback	Low self-efficacy, low health literacy, anxiety towards technology, lack of exposure regarding eHealth, and language barriers
Harnessing mobile technology to support type 2 diabetes self-management among Chinese and Hispanic immigrants: a mixed-methods acceptability study	Yu et al.	2021	Chinese and Hispanic immigrants	Mixed methods design: surveys and semi-structured interviews	Usability, social influence, and family support	Lack of support, complexity, eyesight issues, language barriers, and technology gap
Barriers to digital health services among people living in areas of socioeconomic disadvantage: research from hospital diabetes and antenatal clinics	Freeman et al.	2021	People living in areas of socioeconomic disadvantage	Mixed methods design: surveys combined with interviews	mHealth complementary to face-to-face care, recommendation from a health professional, and national digital infrastructure	Limited technical skills, language barriers, limited health literacy, limited access to technology, costs, and lack of confidence

Longitudinal study						
Title	Author(s)	Year	Target group	Study design	Key facilitators	Key barriers
Use of mobile health applications in low-income populations	Liu et al.	2020	Low-income patients	Prospective study	Implementation of community health workers, technical support, partnering with community organisations, hiring community health workers, translation, and scalability	Lack of digital literacy, limited health literacy, lack of empowerment, historical mistrust of healthcare systems, concerns about privacy and data security, access to technology, and lacking infrastructure

Viewpoint						
Title	Author(s)	Year	Target group	Study design	Key facilitators	Key barriers
It is time to REACT: opportunities for digital mental health apps to reduce mental health disparities in racially and ethnically minoritised groups^b^	Friis-Healy et al.	2021	Racially and ethnically minoritised groups	Viewpoint	Real-world evidence, educating providers and consumers, adaptive interventions, creating for diverse populations, and building trust	Lack of timely effectiveness data, low provider and consumer confidence in intervention quality, lack of provider competency, lack of digital access and literacy, underrepresentation of racially and ethnically minoritised groups in the product development process, the lack of culturally appropriate tailoring, the lack of stakeholder input, and the lack of broader community-partnered implementation plans

^a^ This study (partly) included intervention types not included in the main search (e.g. SMS interventions and video conferencing interventions).

^b^ This study was funded by the Multi-Health Systems-Conners Fellowship in digital health, and one of the authors received research support and/or consulting fees from a number of digital health companies (i.e. Akili Interactive, BehaVR, Limbix, Luoms Labs, OnDosis, Sana Health, and Tali Health). Thus, there could potentially be a conflict of interest. In addition, the factors reported might have less value because they do not come directly from research but from a viewpoint study.

**Table 4.** Overview of key facilitators and barriers and the authors discussing them.

Level	Facilitator/barrier^a^		Source^b^	# of studies	% of studies
**Intrapersonal level**					
(Low) digital literacy		-	[1, 3*, 5, 6*, 7*, 9, 11–13, 15*, 16, 20*]	12/13	92
(Lack of) time		-	[3*, 15*, 16]	3/13	23
(Low) health literacy		-	[7*, 9, 11]	3/13	23
(Dis)Trust		+ -	[7*, 13]	2/13	15
Beliefs about privacy and confidentiality		-	[1, 3*]	2/13	15
**Interpersonal level**					
Human interaction		+ -	[4*, 5, 6*, 7*, 9, 11, 15*]	7/13	54
Healthcare professionals introducing mHealth		+ -	[4*, 6*, 9, 11, 13, 15*]	6/13	46
Trained personnel		+	[3*, 5, 6*, 7*, 13, 15*]	6/13	46
Digital training		+	[3*, 5, 7*, 13]	4/13	31
Social support		+	[12, 15*]	2/13	15
Instructions and support		+	[5, 15*]	2/13	15
**Community level**					
Stakeholder collaboration		+	[1, 4*, 6*, 7*, 13, 15*, 16]	7/13	54
Personnel originated from the community		+	[1, 4*, 6*, 7*, 13]	5/13	39
Cosmopolitanism		+	[6*, 16]	2/13	15
Prioritising		+ -	[3*, 16]	2/13	15
**Ecological level**					
Infrastructure		+ -	[1, 3*, 6*, 7*, 11, 15*, 16, 20*]	8/13	62
Costs		-	[3*, 7*, 11, 15*, 16, 20*]	6/13	46
Policies		+ -	[3*, 4*, 6*, 16]	4/13	31
Reimbursement coverage		+ -	[3*, 4*, 6*]	3/13	23
Governance		+ -	[3*, 6*]	2/13	15
Familiar technology		+	[20*]	1/13	8
**App-specific level**					
Tailoring		+ -	[3*, 4*, 5, 6*, 7*, 9, 12, 13, 15*, 20*]	10/13	77
Usability		+	[5, 7*, 12, 15*, 16]	5/13	39
Visuals		+ -	[1, 7*, 9, 15*, 20*]	5/13	39
Evidence-based		+ -	[4*, 13, 15*, 16]	4/13	31
Language		+ -	[1, 3*, 7*, 9]	4/13	31
Personalisation		+	[3*, 13, 15*, 20*]	4/13	31
Patient-centred design		+ -	[3*, 7*, 13]	3/13	23
Privacy and secure data sharing		+ -	[6*, 13, 20*]	3/13	23
Frequency of reminders		+ -	[15*, 20*]	2/13	15

^a^ A + indicates that it is a facilitator, a - indicates a barrier, and a + - indicates that it can be both a barrier and a facilitator. In the latter case, it is often the case that presence of the factor is a facilitator, and absence of the factor is a barrier.

^b^ Asterisks (*) were used to indicate when this was a literature review based on multiple studies so that value could be placed on the number and type of source citations.

## Results

### Selection and characteristics of sources of evidence

**Figure 1.**
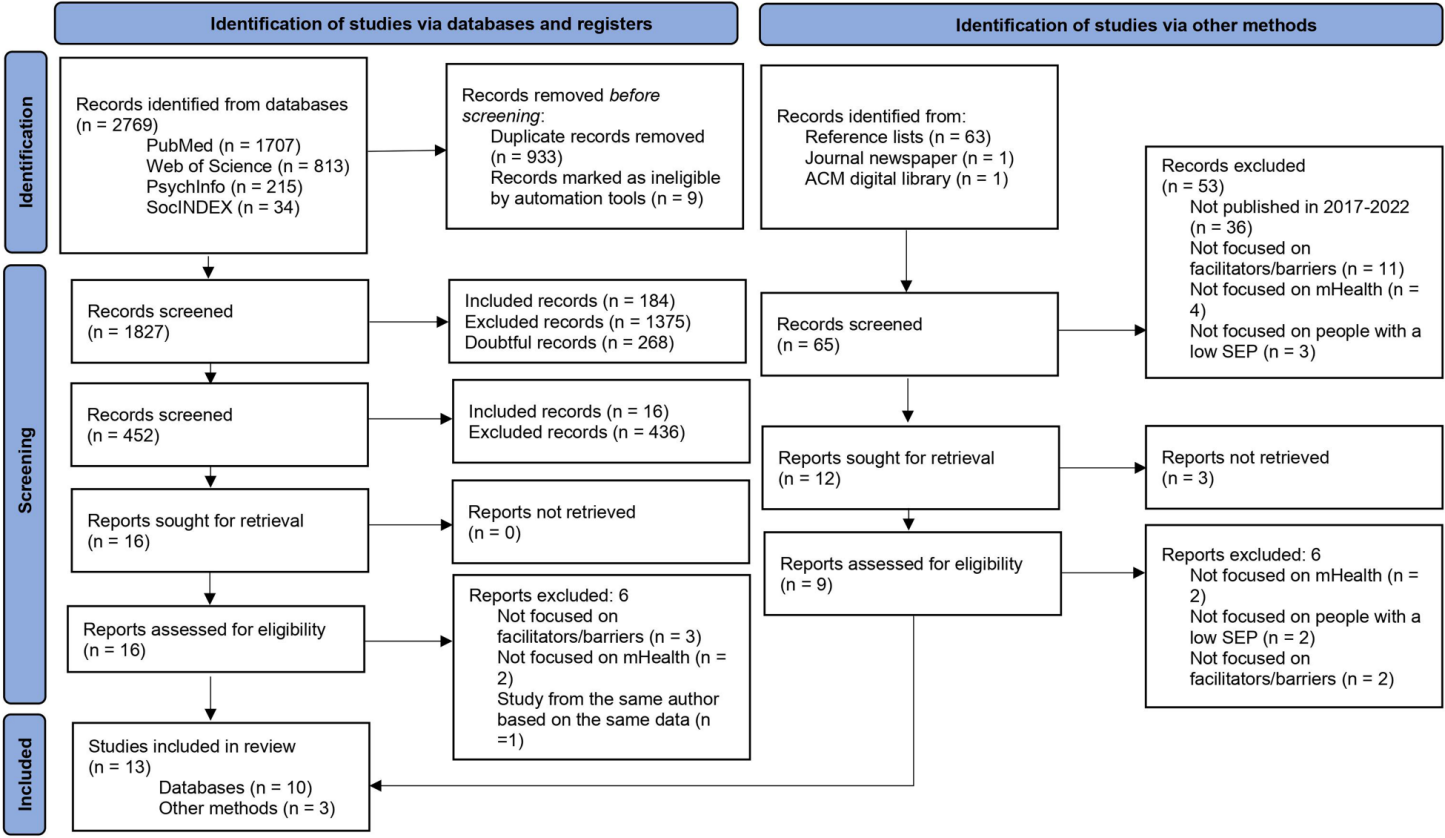
PRISMA 2020 flow diagram for new systematic scoping reviews.

Of the 1827 papers that emerged from the databases, 10 were found to be suitable for inclusion in the current study. Three more studies were included from the reference lists and other sources, resulting in a total scope of 13 articles. The full PRISMA flow diagram can be found in Figure 1.^19^

Of the thirteen articles, there were three scoping reviews,^6,7,17^ three systematic reviews^3,4,15^ and four mixed method studies.^5,9,11,13^ Moreover, one article included an RCT,^18^ one article was a viewpoint^14^ and one article was a prospective study.^1^

The selection included three articles focused on low-income people or countries,^1,3,18^ five articles focused on ethnic minorities^4,6,7,13,14^ and five articles focused on people with a low socio-economic status in general.^5,9,11,15,17^ No articles were included that focused specifically on people with low levels of education. A full overview of the target group, type of study and key barriers and facilitators of each study for the end user can be found in Table 3.

### Results of individual sources of evidence

See Table 3.

### Synthesis of results

In the overview below all facilitators and barriers are outlined using the classification of Stowell and Lyson,^15^ adapted to the current study as described in the methods section.

### Intrapersonal

As for the individual themself (i.e. the end user), low literacy appeared to be a barrier to adopting mHealth. This could either be a low health literacy^7,9,11^ or a low digital literacy.^1,3,5,7,9,11,13–15,17,18^ Regarding the latter, Kruse, Betancourt^3^ and Steinman and van Pelt^18^ even spoke of a technology gap (i.e. the difference between owning a device and mastering it), highlighting the importance of an mHealth application that supports the most vulnerable populations^18^ and all levels of digital literacy.^6^

Moreover, the beliefs of the end user appeared to be an important factor in mHealth adoption. One such belief is distrust towards the governments, unknown organisations or the healthcare system, which could be caused by historic traumas, stigmas or previous experiences.^7,14^ Distrust is seen as a barrier that accounts for low engagement.^14^ On the other hand, generating trust can also be a facilitator of mHealth adoption.^7^ Moreover, another important belief included concerns about privacy and confidentiality of information, which forms a barrier to mHealth adoption.^1,3^ According to Faber and Al-Dhahir^9^ it is, therefore, crucial to align mHealth to these attitudes to enhance adoption.

Finally, another intrapersonal barrier is the lack of time people perceive to invest in mHealth.^3,17,18^ Due to psychosocial stressors such as a busy work and family life,^7^ worries about finances, or the working environment,^17^ people may be less willing and/or able to invest time in mHealth. It is therefore important that users understand the relative advantage of using mHealth so as to start seeing it as a priority.^18^

### Interpersonal

On an interpersonal level, the focus lies on the influence others exert on the individual end user, such as family, friends and carers.

Despite the fact that we excluded the articles on mHealth apps that involved human interaction, the included articles revealed that, from the perspective of the individual with a low SEP, human contact is inevitably associated with the adoption of mHealth applications. Thus, human interaction with healthcare professionals appeared to be an important facilitator of mHealth adoption.^4–7,9,11,17^ Health professionals could be lay health workers, community health workers, peers, nurses, physicians and researchers.^4^ Human interaction with these health professionals could be both integrated into mHealth and provided face-to-face to establish a trustful relationship with the individual.

In this regard, healthcare professionals could offer technological support.^4,6,7,9^ This is especially important when delivering a new application because it was found that end users do often not know how to use the technology.^11^ Hence, digital training to teach the end user how to use mHealth is found to be another facilitator.^3,5,7,14^ Not only does this help with the performance, but it also increases the acceptability of mHealth. Digital training and support could be provided by family and friends, as support from family or friends is also deemed important as it can provide social, emotional and instrumental support.^13,17^ However, relatives of people with low SEP may not always be capable of doing this. In that case, health personnel can also provide assistance.

In addition, healthcare personnel could serve as an intermediary to introduce mHealth to the vulnerable individuals as they can enhance trust and make people aware of the available mHealth solutions, facilitating their adoption.^4,6,9,11,14,17^ This is important as it was found that people with a low SEP are often unaware of mHealth opportunities.^7^

Hence, the combination of mHealth and face-to-face human interaction is a great facilitator for the mHealth adoption.^11^ This combination of personal contact with healthcare professionals and mHealth is called ‘blended care’.^17^ Blended care enhances engagement^17^ and enhances trust with technology (and specifically new mHealth modalities).^6^

However, for care providers to interact with end users, the care providers should be well informed and trained on both technology use, the corresponding etiquette,^3,6,17^ and cultural safety.^7^ This enables care providers to make informed choices and recommendations for end users (e.g. on which apps to use)^14^ and become more trustworthy.^5^ That way, the staff should also be available and able to offer technical support to help access and utilise mHealth applications,^6^ including detailed instructions and long-term support.^5,17^

The aforementioned interpersonal facilitators were proven to be successful in the study by Liu, Astudillo,^1^ in which community health workers were employed to offer technical support, provide trust and a supportive relationship and answer questions. However, it should be noted that a shortage of healthcare staff can act as a barrier.^6^

### Community

In addition to the healthcare professionals, other stakeholders have been identified in the literature who can be categorised under the community level (i.e. the physical and social environment including the influence on the individual of societal relationships, external companies and residential areas). The main stakeholders mentioned in the studies were the government, medical companies, insurance companies, mHealth developers and researchers.

With regard to these stakeholders, several facilitators were mentioned in the literature. First, the stakeholders need to see adapting mHealth to the low SEP group as a priority in order to be committed and involved.^3,18^ However, when mHealth is not prioritised, this forms a barrier for mHealth implementation.^18^

Second, stakeholder collaboration is an important facilitator of mHealth adoption.^1,6,7,17,18^ Important collaborations are those between developers and national organisations,^6,18^ such as governments,^18^ health insurers,^17^ the technology sector^18^ and between local organisations and parties, such as non-profit organisations, communities and researchers.^1,7^

However, for stakeholder collaboration to lead to adoption, a cultural bridge is needed between the low SEP individual (i.e. the end user) and stakeholders such as companies,^7^ forming another facilitator. A cultural bridge designates someone from the target group who is the liaison between the different parties that are involved. Even more desirably, intermediaries from the target group are engaged, trained and employed as researchers, policymakers, educators, providers and developers^1,4,6,7,14^ by which staff shares the cultural background or primary language with the mHealth's user^1,7^ and thus serves as a cultural bridge.

According to Steinman and van Pelt^18^ and Bailey and Gurgol,^6^ these stakeholders should then come together in a cosmopolitan system and workflow, which is another facilitator. This means that all existing healthcare networks, systems and providers are linked and work together. These elements leverage each other's expertise and experiences. This, in turn, makes working with the system easier and more efficient.

### Ecological

The ecological level looks at the influence of the combination of previously mentioned factors along with the influence of laws, regulations and policies.

On an ecological level, mostly barriers were mentioned in the literature. One of the main barriers of adopting and using mHealth is a lack of infrastructure.^1,3,6,7,11,15,18^ Here, infrastructure mostly refers to the facilities around mHealth, such as access to the Internet and a mobile device. Hence, as reported in the studies, a lack of service or Wi-Fi is an experienced barrier.^1,7,17,18^ Therefore, it is necessary to facilitate the adoption by ensuring a good national digital infrastructure where everyone has a mobile device and is equipped with service and Internet.^11^ One way to realise this is by increasing the number of hotspots with access to free Wi-Fi.^1^ Another facilitator is when people have access to a mobile device on which they can use mHealth,^1,3,6,11,15,18^ that is technically suitable^7^ and familiar to the user.^15^

However, mobile phones and phone services appeared to be unaffordable for people with a low SEP, forming a huge barrier.^3,7,11,15,17,18^ Therefore, reimbursement coverage is necessary. Partnerships should be established with both governments and non-governmental organisations to account for funding, leadership and the required infrastructure.^3,4,6^ Partnering with the private sector (e.g. equipment providers) was also recommended to ensure that the number of people who possess a phone continues to increase.^3^

Thus, permanent changes in policies and regulations are necessary to ensure that there is high mobile phone coverage among people with a low SEP along with the alignment of mHealth with international and national plans.^6,18^ A lack of policies was a commonly cited barrier to mHealth integration.^3,4,6,18^ Hence, within these policies, the telephone service should be prioritised in order to ensure access to mHealth. All in all, this should be provided with strong governance.^3,6^

### App specific

Finally, a number of application-specific factors were mentioned that either promote or hinder the adoption and use of mHealth.

A patient-centred design is an important facilitator.^6,7^ By this, people who are most at risk of health concerns and who face barriers to healthcare engagement should be engaged in the development of mHealth^14,17,18^ by providing feedback on the usability and feasibility of the technology to meet community needs.^6,7^ Thus, by involving the target group, insight should also be gained into their differing life situations, motivations, healthcare needs and expectations of the target group.^9^ Therefore, inadequate involvement of the target group in the development stage can also act as a barrier.^14^ Pilot testing is therefore also a recommendation, whereby designing mHealth could be seen as an iterative process in which feedback from the users is processed and the app is adapted and redesigned within every stage^7,17^ and as technology advances.^3^

However, this co-creation should go hand in hand with research to provide strong and qualitative evidence on mHealth use which can be carried through into the design at all stages of development.^3,14,17,18^ Evidence-based mHealth, in turn, is more effective and provides more engagement.^4^ In addition, providing users real-time insight into the data could increase or enhance trust and helps to improve the application in a timely manner.^14^ Within this data, fidelity, adoption, uptake, usefulness, usability and costs are particularly important.^9,14^ However, a lack of timely effectiveness data can be a barrier.^14^ Therefore, through such research, evidence-based guidelines could be made to support the creation of mHealth for people with a low SEP.^7^

One of the evidence-based design guidelines would be that mHealth should be easy to use and understand.^5,7,13,17,18^ Hence, the software, hardware and service should be simple, easy to use, readily available and reliable.^5,7,17^ As proposed by Faber and Al-Dhahir,^9^ one way to integrate this is with interactive animated computer characters, which use non-verbal cues such as simple speech and hand gestures, by which social contact is simulated and understanding of the content is simplified. This might also engage through variation and gamification elements, as recommended by Stowell and Lyson.^15^ Moreover, Agachi and Bijmolt^23^ stated that amongst people with a low SEP an application is preferred over a website.

Complexity could also be reduced by paying attention to language use^5,7,9^ as low literacy and language barriers were found to be important barriers to mHealth adoption.^3,9,11,13,17^ Steinman and van Pelt^18^ found that voice messages could not resolve this low literacy. However, by matching the language use between users and service providers and offering the possibility of translating written and audiovisual material, complexity could be reduced.^1,7^ Therefore, the language used should be friendly and not too coercive, avoiding medical terms.^5^

In addition, using visuals, such as audio, images or videos is another facilitator of using mHealth.^1,7,9,15,17^ Visual elements are found to be more appealing and easier to recall than text. Thus, through their use, the barrier of low literacy could be minimalised.^15^ The study by Faber and Al-Dhahir^9^ also highlighted the relevance of visual feedback. However, attention will need to be paid to the amount of visuals, as an excess of (information or) visuals can in turn become a barrier.^17^

Another facilitator of mHealth is the application of tailoring^3–7,9,13–15,17^ while a lack of tailoring can also be a barrier.^14^ Within tailoring, the application and the information within are adapted to fit the characteristics, needs and preferences of the whole target group.^4,5,14^ Tailoring should be based on the cultural context (i.e. cultural tailoring): norms and attitudes of the culture (i.e. subjective culture); the behavioural preferences; and cultural values and expectations of the community.^6,7,14^ Furthermore, the personal, social and economic context should be taken into account.^4^ Next to cultural tailoring, content can also be tailored at the medical level by personalising the medical message or at the literacy level by tailoring the content to the level of literacy of the user.^9^

Moreover, personalisation is a facilitator for the uptake of mHealth applications. Whereas tailoring ensures that the application is developed based on cultural, personal, social, economic and medical aspects of the target group, personalisation ensures that each individual can set up the application to his or her own liking. This could be done in several ways. Frequent (but adequate) delivery of reminders was deemed to be important.^15,17^ Kruse and Betancourt^3^ and Stowell and Lyson^15^ add to this by discussing the importance of the option to personalise the timing of the reminders based on mobile network fluctuations, critical timing for the intervention and the availability of the end user. However, the application itself and its messages could also be personalised based on individual characteristics (e.g. gender, language preferences), socio-economic and socio-cultural preferences.^17^

Finally, for individuals of low SEP to use mHealth, it is also essential that confidential protected health information sharing and preserving privacy is guaranteed.^6,15^ The confidential sharing of personal information is therefore seen as a facilitator. When this is not sufficiently ensured, it is a barrier to mHealth adoption.^6^ The corresponding statements about data security should therefore be clear and easily interpretable.^14^

## Discussion

### Summary of evidence

This scoping review provided an overview of the facilitators and barriers to the adoption of mHealth applications among people with a low SEP. The most prevalent factors were the infrastructure, stakeholder collaboration, human interaction, tailoring and digital literacy. Herein, the infrastructure could be both a facilitator as a barrier, as well as human interaction and tailoring. Stakeholder collaboration facilitates the mHealth adoption, whereas digital literacy is a barrier of mHealth adoption. The findings illustrate the value of the adapted categorisation of Stowell and Lyson^15^ consisting of five levels of facilitators and barriers that influence adoption. Herein, the end user and the application, but also external persons and parties and the environment are considered.

It should be noted that many factors are assumed to be interrelated. For example, many app-specific factors are a result of low digital literacy (e.g. the recommendation for an easy-to-use application), which in turn makes digital training and support necessary. Also, the lack of policies is indirectly related to the lack of infrastructure and reimbursement. As discussed by Steinman and van Pelt,^18^ partnerships with external organisations are necessary to secure funding, governance and infrastructure. Infrastructure and digital training are, in turn, necessary to engage the low SEP population. Moreover, among some levels, there are many commonalities and interrelationships, such as between the interpersonal and community level. It is therefore important to consider not only the five levels individually but the entire mHealth environment with interrelating factors as well.

**Figure 2.**
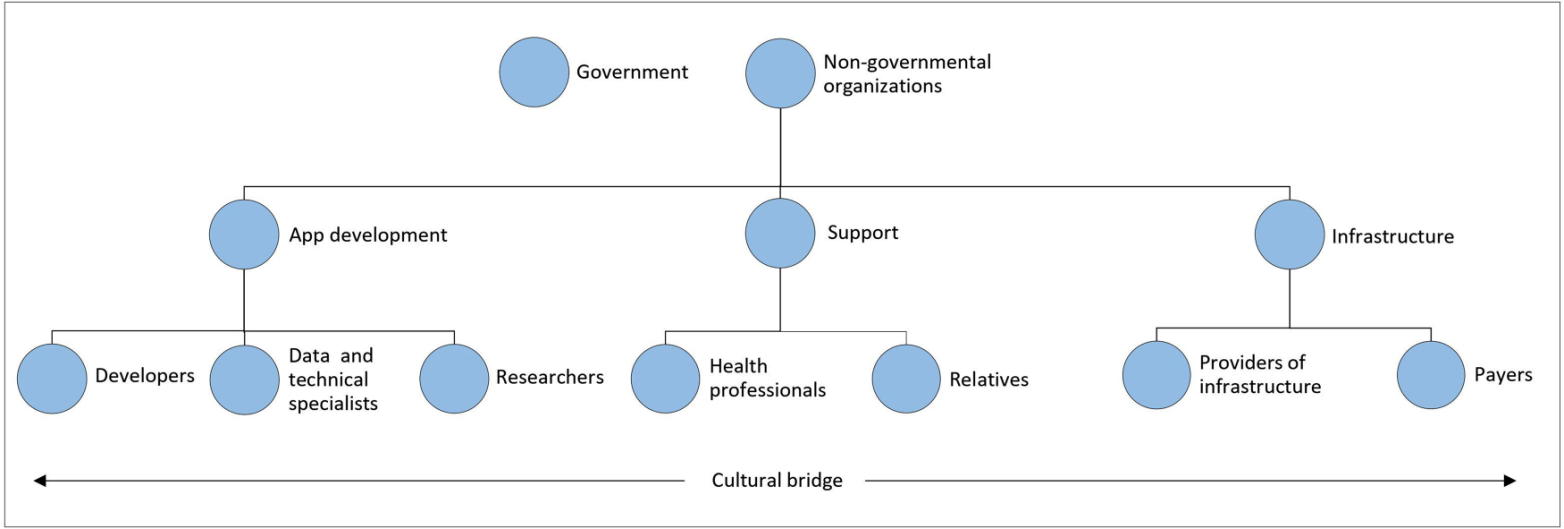
Cosmopolitan system of mHealth stakeholders based on the current study.

It may therefore be concluded that within the mHealth environment there is a need for a well-structured cosmopolitan system. Hence, a clear cosmopolitan system of cooperating stakeholders will need to be set up (see Figure 2 for an outline of the proposed cosmopolitan system). For example, health professionals will need to be trained technologically and culturally, and can then provide personal contact, trust, digital training and support to the mHealth user. Training in the cultural field can be provided by people from the target group who serve as a cultural bridge between the non-governmental organisations and end users. In addition, data and technical specialists can take up the technological training, ensuring that privacy is guaranteed and that data is managed. Next, governmental and non-governmental organisations will have to take care of the digital infrastructure, reimbursement coverage and the policies in which this is established. Finally, the app-specific requirements and demands can be applied in the applications by developers, who should base their decisions on a patient-centred design and qualitative and up-to-date research.

In addition, special attention is needed for the relationship between traditional (offline) care and digital care. On the interpersonal level, there appeared to be a need for personal contact with carers. Specifically, carers introducing people to mHealth applications and providing technological support alongside their use of mHealth appeared to be a facilitator. However, the scope of the current study was limited in terms of researching support regarding mHealth adoption as we excluded studies that address the opinion or evaluation of carers, medical applications focused on carers or applications that involve carers. Moreover, relatively little is known in the literature about how the support around mHealth might best be designed. Hence, a greater focus on researching and designing the support regarding mHealth usage could produce necessary insights to facilitate mHealth adoption. With this, careful consideration of feasibility is required. Carers providing extra personal contact might not be feasible because of the high workload that healthcare personnel regularly face. It should therefore be investigated whether this support could also be provided by key people other than the carers. These could, for example, include welfare workers, support staff in the public library or in a community organisation, volunteers from one's own social network, or assistants of general practitioners.

Besides addressing autonomous applications, the current study focused on the population with low SEP. This means that the current study offers insights into the adoption of autonomous mHealth applications among people with low SEP. Overall, the insights of the current study largely correspond to the findings of the Dutch National Institute for Public Health and the Environment (RIVM),^24^ who state that there are certain preconditions for the technology to be used optimally to achieve a certain goal and to fit its users. These preconditions include well-functioning technology, connected systems, the ability to exchange information quickly and securely and the careful integration of digital applications into the care process. In addition to the technical aspects, digital skills, mutual trust, leadership and good cooperation are also mentioned as essential prerequisites.

Moreover, similarities have been found with the theoretical framework for Non-adoption, Abandonment, and Challenges to the Scale-Up, Spread, and Sustainability (NASSS). The NASSS framework consists of seven domains: condition; technology; value proposition; adopters; (healthcare) organisation(s); wider system and embedding and adaption over time.^25^ These domains map possible areas of complexity important for the non-adoption and abandonment of technologies by individuals. Herein, the ‘condition’ domain equals the low SEP group aimed at in the current study. The ‘technology’, ‘adopters’, ‘organisational’ and ‘wider system’ domains refer respectively to the app-specific, intrapersonal, interpersonal, community and ecological levels from the classification of Stowell and Lyson.^15^ Moreover, the results illustrated that both the end user and other stakeholders should prioritise mHealth in order to stimulate mHealth adoption. This is explicitly reflected in the domain ‘value proposition’ in the NASSS framework. Finally, it was found that mHealth should be developed within an iterative process in order to remain supply-side and demand-side value. This is what the ‘embedding and adaptation over time’ domain of the NASSS framework focuses on. Hence, it can be concluded that the NASSS framework provides confirmation for the classification of the five levels, the successive demand for a cosmopolitan system and the insights related to this within the current study. It is noteworthy, however, that the NASSS framework did not serve as the basis of any of the included studies. Within the study by Steinman and van Pelt,^18^ the RE-AIM QuEST mixed methods framework was used for the evaluation of the programme. In addition, the study by Yu and Wu^13^ used the Unified Theory of Acceptance and Use of Technology (UTAUT) framework and the study by Eggink and Hafdi^5^ used the attitude, social influence and self-efficacy (ASE) model, both focused on (use) behaviour. The other included studies did not explicitly use a theoretical framework. Hence, it would be valuable to more frequently set up theoretically framed studies within different contexts and to link research findings to theoretical models in order to obtain a clear and scientifically based insight into the facilitators and barriers in mHealth adoption.

### Limitations and future research

#### Limitations

The first limitation to our review was that many studies use a different definition of mHealth. The current study has conceptualised mHealth as the applications that can be used independently and autonomously on a mobile device (e.g. smartphone, tablet). Applications including video conferences with external persons such as health professionals, for example, were therefore excluded. However, some articles understand mHealth to include a short message service (SMS), text messaging, calls and mobile apps (Kruse et al., 2019). An example is the included study by Steinman and van Pelt^18^ which focused on mHealth and eHealth use for diabetes and hypertension management amongst hard-to-reach populations in which SMS interventions and video-conferencing interventions were used. The latter study was relevant to include because of the facilitators and barriers mentioned for autonomous applications, but the differences in operationalisations made it more difficult to conduct a clear search in the databases in the current study. Hence, for follow-up research, it would be valuable to distinguish within mHealth between studies that use movement interventions such as motion sensors, VR or AI, studies that use interactive elements such as SMS interventions, chat interventions or video conferencing and autonomous applications that can be used by a single user and without additional technologies. Insights into the facilitators and barriers of adoption should be obtained for all these types of interventions in order to find indications for optimising the adoption of each type of intervention according to these factors.

Second, it should be noted that the facilitators of mHealth are often related to the barriers of traditional healthcare. For example, costs are different for mHealth than for traditional care. For traditional care, the patient has to visit healthcare staff, which can lead to high costs. However, for people with a low SEP, it is not always possible to visit healthcare facilities because they cannot afford to take time off work for a hospital visit, do not have money to cover transportation or parking costs or are not skilled enough to reach the location by public transportation. In this regard, mHealth has the potential to overcome the barriers of traditional healthcare and thereby improve access to care. Thus, the relationship between mHealth and traditional care (and the costs thereof) has an impact on the potential and the importance of ensuring appropriate reimbursement. As can be concluded from the scoping review, mHealth should therefore not be seen as an independent system or a replacement for the current system, but rather as a part of the healthcare system and complementary to traditional healthcare to be able to reach this full potential.

Third, quantitative support for the strength and evidence of the factors in the included studies was often lacking in the included articles and so the genuine influence of the factors could not be proven in our scoping review. Though, it should be noted that this was also not the aim of this review in contrast to meta-analyses and systematic reviews. Yu and Wu^13^ did use statistical tests (i.e. *t*-tests and Chi-squared tests) to account for sociodemographic details and technology adoption and Freeman and Fisher^11^ explained the role of the healthcare specialist through quantitative evidence. However, of the 13 included studies, no studies used statistical tests to provide robust evidence for the facilitators and barriers of mHealth adoption. Thus, while there has been qualitative research on the factors that promote or hinder mHealth adoption among people with a low SEP, there is no quantitative evidence to support this based on the correlation analyses of other statistical tests. Hence, depth should be sought in future research by adding quantitative evidence to the studies.

#### Recommendations

Following the limitations, there are three recommendations that are specifically aimed at follow-up research.

First, despite the fact that people with low levels of education have less access to social, informational and material resources related to health and well-being^15^ and were found to make less use of health-related technology,^13^ no articles could be included that have been published within the last five years that focused specifically on people with lower levels of education. Thus, it would be a natural progression of this work to conduct future research specifically on the factors that promote or hinder mHealth adoption among end users with a lower educational attainment.

Second, the current study also revealed a clear need for human interaction and support to increase mHealth adoption. However, studies focusing on support around mHealth were not included in the original search because the focus was on autonomous mHealth applications. Therefore, to extend the insights of the current study it is important to conduct an overview study focusing specifically on support related to mHealth to gain more insight into the requirements around this concept. Likewise, it would be relevant to conduct research among a broader range of vulnerable groups to provide insight into mHealth adoption from a broader perspective.

Third, within the group of people with a low SEP, subgroups may be assignable. According to Latulippe and Hamel,^12^ the facilitators and barriers differentiate between people with different ethnicity, income and level of education. Therefore, within the current study, the facilitators and barriers were looked upon for vulnerable people with low income, low education level or an ethnic minority status. However, Latulippe and Hamel^12^ further suggest that the factors might also differ according to demographics such as age and gender. Within the literature that we included on lower SEP groups there has been little attention paid to these differences between, for example, men and women, healthy and unhealthy people, or young and elderly people. It would therefore be relevant for follow-up research to study within this low SEP population whether there are differences among different subgroups.

## Conclusions

Thirty factors were found to facilitate and/or impede mHealth adoption among people with a low SEP along the adapted categorisation of Stowell and Lyson.^15^ Many of the factors along the five levels of categorisation were assumed to be interrelated. Therefore, a well-structured cosmopolitan system of stakeholders has been recommended. In this way, healthcare networks, systems and providers can work together easily and efficiently. Of particular interest here is the relationship between traditional (offline) care and digital care as the current study revealed that face-to-face contact is a prerequisite of mHealth adoption among people with low SEP. In the end, this study underlined the value of the categorisation by Stowell and Lyson. Moreover, knowledge gaps that may stagnate efforts to increase mHealth adoption have been brought to light. Thus, future research is recommended on the design and allocation of support around mHealth. In addition, it is important to gain an understanding of the differences between particular low SEP subgroups (e.g. men compared to women or the elderly compared to the young). Next, studies on mHealth adoption should be substantiated with theoretical models and scientific quantitative underpinnings. This will allow a better understanding of the adoption of mHealth by people with a low SEP, which in turn may lead to improved access, support and user design to facilitate a higher adoption and accordingly reduced health disparities.

## Abbreviations

Low SEP: Low Socio-Economic Position
mHealth: mobile health applications
WHO: World Health Organization

## Notes

1. Preferred Reporting Items for Systematic reviews and Meta-Analyses extension for Scoping Reviews.

## Appendix A: PRISMA-ScR**^1^** checklist.

**Table:**

Section	Item	PRISMA-ScR checklist item	Reported on page #
TITLE			
Title	1	Identify the report as a scoping review.	1
ABSTRACT			
Structured summary	2	Provide a structured summary that includes (as applicable): background, objectives, eligibility criteria, sources of evidence, charting methods, results, and conclusions that relate to the review questions and objectives.	1
INTRODUCTION			
Rationale	3	Describe the rationale for the review in the context of what is already known. Explain why the review questions/objectives lend themselves to a scoping review approach.	1
Objectives	4	Provide an explicit statement of the questions and objectives being addressed with reference to their key elements (e.g. population or participants, concepts, and context) or other relevant key elements used to conceptualise the review questions and/or objectives.	2
METHODS			
Protocol and registration	5	Indicate whether a review protocol exists; state if and where it can be accessed (e.g. a Web address); and if available, provide registration information, including the registration number.	3
Eligibility criteria	6	Specify characteristics of the sources of evidence used as eligibility criteria (e.g. years considered, language, and publication status), and provide a rationale.	3
Information sources^a^	7	Describe all information sources in the search (e.g. databases with dates of coverage and contact with authors to identify additional sources), as well as the date the most recent search was executed.	3
Search	8	Present the full electronic search strategy for at least 1 database, including any limits used, such that it could be repeated.	4
Selection of sources of evidence^b^	9	State the process for selecting sources of evidence (i.e. screening and eligibility) included in the scoping review.	5
Data charting process^c^	10	Describe the methods of charting data from the included sources of evidence (e.g. calibrated forms or forms that have been tested by the team before their use, and whether data charting was done independently or in duplicate) and any processes for obtaining and confirming data from investigators.	5
Data items	11	List and define all variables for which data were sought and any assumptions and simplifications made.	5
Critical appraisal of individual sources of evidence^d^	12	If done, provide a rationale for conducting a critical appraisal of included sources of evidence; describe the methods used and how this information was used in any data synthesis (if appropriate).	N/A
Synthesis of results	13	Describe the methods of handling and summarising the data that were charted.	5
RESULTS			
Selection of sources of evidence	14	Give numbers of sources of evidence screened, assessed for eligibility, and included in the review, with reasons for exclusions at each stage, ideally using a flow diagram.	5
Characteristics of sources of evidence	15	For each source of evidence, present characteristics for which data were charted and provide the citations.	5
Critical appraisal within sources of evidence	16	If done, present data on critical appraisal of included sources of evidence (see item 12).	N/A
Results of individual sources of evidence	17	For each included source of evidence, present the relevant data that were charted that relate to the review questions and objectives.	6-9
Synthesis of results	18	Summarise and/or present the charting results as they relate to the review questions and objectives.	12
DISCUSSION			
Summary of evidence	19	Summarise the main results (including an overview of concepts, themes, and types of evidence available), link to the review questions and objectives, and consider the relevance to key groups.	15
Limitations	20	Discuss the limitations of the scoping review process.	16
Conclusions	21	Provide a general interpretation of the results with respect to the review questions and objectives, as well as potential implications and/or next steps.	18
FUNDING			
Funding	22	Describe sources of funding for the included sources of evidence, as well as sources of funding for the scoping review. Describe the role of the funders of the scoping review.	18

^a^ Where *sources of evidence* (see second footnote) are compiled from, such as bibliographic databases, social media platforms, and Web sites.

^b^ A more inclusive/heterogeneous term used to account for the different types of evidence or data sources (e.g. quantitative and/or qualitative research, expert opinion, and policy documents) that may be eligible in a scoping review as opposed to only studies. This is not to be confused with *information sources* (see first footnote).

^c^ The frameworks by Arksey and O’Malley^6^ and Levac et al.^7^ and the JBI guidance^4,5^ refer to the process of data extraction in a scoping review as data charting*.*

^d^ The process of systematically examining research evidence to assess its validity, results, and relevance before using it to inform a decision. This term is used for items 12 and 19 instead of “risk of bias” (which is more applicable to systematic reviews of interventions) to include and acknowledge the various sources of evidence that may be used in a scoping review (e.g. quantitative and/or qualitative research, expert opinion, and policy document).
